# Ostéome ostéoïde du talus gauche chez l'enfant

**DOI:** 10.11604/pamj.2013.14.33.1530

**Published:** 2013-01-23

**Authors:** Abdelhalim Mahmoudi, Mouhcine Bendahou, Mohamed Rami, Khalid Khattala, Afaf Amarti, Bouabdallah Youssef, My Abderrahmane Afifi

**Affiliations:** 1 Service de Chirurgie Pédiatrique CHU Hassan II de Fès, Maroc; 2 Service d'Anatomie Pathologique CHU Hassan II de Fès, Maroc

**Keywords:** Ostéome ostéoïde, Talus, enfant, Osteoid osteoma, Talus, children

## Abstract

La localisation de l'ostéome ostéoïde au niveau de la cheville est rare. Nous rapportons le cas d'une jeune fille qui présentait un ostéome ostéoïde du talus gauche dont le diagnostic a été évoqué par la TDM et dont l'exérèse a été réalisée à ciel ouvert. L'examen histologique de la pièce a confirmé le diagnostic. Les suites opératoires ont été simples avec une disparition complète des douleurs sans recidives après un recul de 3 ans.

## Introduction

L'ostéome ostéoïde (OO) a été décrit pour la première fois par Jaff [1] en 1935. Il s'agit d'une tumeur ostéoblastique bénigne qui comporte une petite lésion centrale charnue très vascularisée ostéoïde et immature (le nidus) entourée d'une ostéocondensation réactionnelle. Elle est découverte le plus souvent chez l'adolescent et l'adulte jeune. Cette lésion est située préférentiellement au fémur proximal. Les localisations à la cheville sont moins fréquentes et au talus elles représentent seulement 5 à 8% des cas [2]. Nous rapportons le cas d'un ostéome ostéoïde du talus gauche chez une adolescente de 12 ans, ayant consulté pour douleurs inflammatoires nocturnes de l'arrière pied gauche évoluant depuis quatre mois.

## Patient et observation

**Figure 1:**
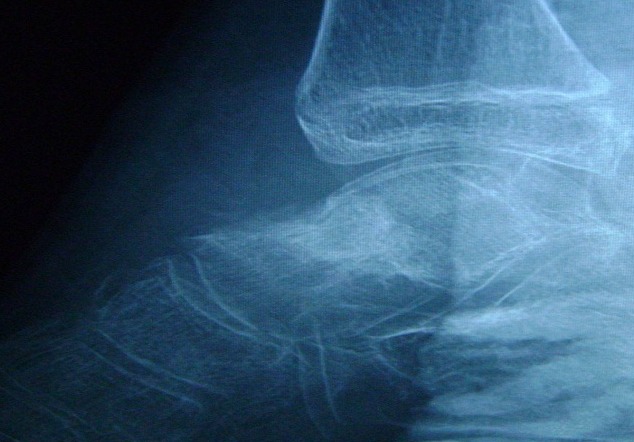
Radiographie de la cheville de profil: image de condensation sur le talus

**Figure 2:**
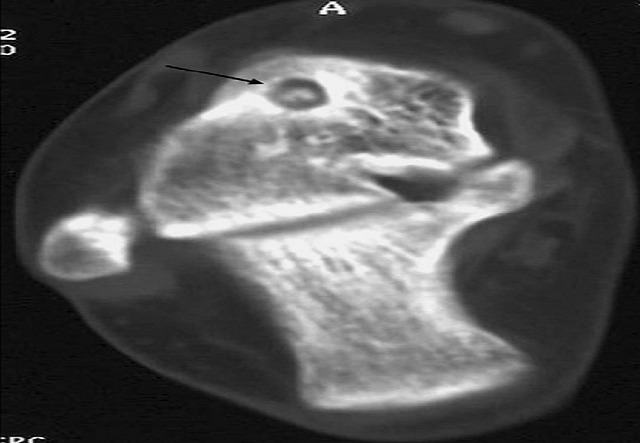
TDM cheville Image hypodense siégeant sur le talus, entourée par un anneau périphérique hyperdense

L. Z âgée de 12 ans, a été admise dans notre service pour des douleurs chroniques de la cheville droite remontant à 4 mois. Cette douleur augmente progressivement d'intensité, mais sans facteurs déclenchants, notamment traumatique. Cette douleur avait un caractère inflammatoire. Elle n'avait pas, par ailleurs, de limitation des amplitudes articulaires de la cheville, ni d'autres signes associés. L'examen de la cheville montrait une cheville globalement douloureuse, sans point douloureux hyperalgique particulier. Les mobilités articulaires, étaient normales. La radiographie de la cheville de profil (Figure 1) montrait un interligne tibiotalien normal conservé, l'analyse fine du cliché de profil a objectivé une image de condensation sur le talus. Une tomodensitométrie de la cheville (Figure 2) a montré une image hypodense siégeant sur le talus, entourée par un anneau périphérique hyperdense. Le bilan inflammatoire était normal.

**Figure 3:**
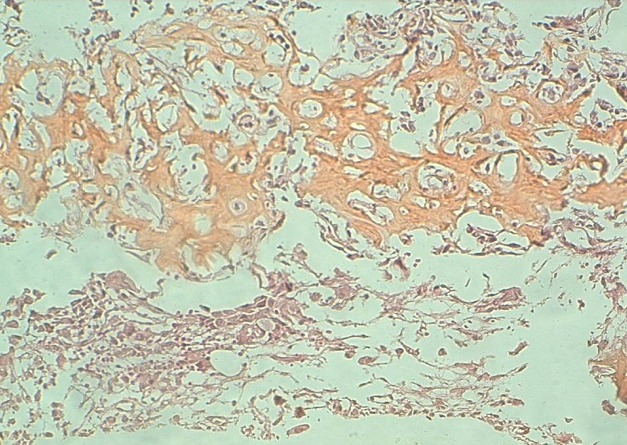
Aspect histologique: Osteome osteoide. HES x200: Il s'agit tantôt d'un tissu fibro-vasculaire fait de capillaires à paroi régulière, de fibroblastes et de rares éléments inflammatoires, tantôt de travées ostéoïdes grêles bordées d'ostéoblastes réguliers sans mitose

Le patient a été opéré à ciel ouvert, par une voie d'abord antérieure, ce qui a permis de mettre en évidence une lésion sphérique, de siège sous périostée, à la face antérieure du talus, L'excision de cette lésion a été faite à la curette. L’étude histologique de la pièce d'exérèse a retrouvé un ostéome ostéoïde du talus (Figure 3). La patient est asymptomatique et n'a pas présenté de récidive tumorale avec un recul de 3 ans.

## Discussion

L'OO est une tumeur osseuse bénigne, relativement fréquente (12% des tumeurs osseuses bénignes). Il touche essentiellement le grand enfant et l'adolescent avec une prédominance masculine [3]. L'atteinte se fait préférentiellement sur la diaphyse d'un os long, notamment sur le fémur et le tibia [4]. La localisation au niveau du pied est beaucoup plus rare et représente 2 à 10% des cas. Au pied, l'atteinte talienne est fréquente [5].

La douleur est le maître symptôme. Cette douleur est très intense, très localisée à recrudescence nocturne. Elle cède habituellement aux anti-inflammatoires non stéroïdes [4].

Sur les radiographies standards, l'image évocatrice de l'OO est une lacune de petite taille toujours inférieure à 1,5 cm, intracorticale, entourée par un ostéosclérose importante. Le nidus peut contenir des calcifications traduisant l'ossification de la matrice ostéoïde dans les lésions anciennes [4].

La TDM en coupes fines jointives constitue la modalité d'imagerie la plus fiable dans le diagnostic d'OO particulièrement quand la lésion siège dans des zones anatomiques complexes et d'analyse difficile sur les clichés standard [5]. La sémiologie tomodensitométrique de l'OO est très évocatrice. L'image du nidus est classiquement une petite hypodensité à contours nets présentant dans 50% des cas une calcification le plus souvent centrale, à contours réguliers [6]. La TDM joue également un rôle important dans le repérage préopératoire des OO avec le développement des résections percutanées [5]. En IRM, la lésion intraosseuse apparaît en hyposignal en T1 et en hypersignal en T2 se réhaussant après injection de gadolinium. La tomodensitométrie.

La scintigraphie est indiquée devant un tableau évocateur alors que les radiographies standards sont interprétées comme normales. Elle montre presque toujours une hyperfixation caractéristique, très localisée [6, 7].

Le traitement de l'ostéome ostéoïde consiste en une résection “en bloc”, emportant la totalité du nidus. Le traitement doit répondre à deux impératifs: réaliser une exérèse complète de la lésion afin d’éviter les récidives [8] et ne pas réséquer de façon trop large au risque d'entraîner une fragilité du segment osseux ou de provoquer des troubles de la croissance chez l'enfant.

Initialement, le traitement chirurgical a été décrit à ciel ouvert. Certains auteurs ont proposé une résection arthroscopique, notamment pour les localisations au talus [9, 10] Dans notre observation, le traitement à ciel ouvert a été facile vue la localisation superficielle de la lésion.

La technique du forage percutané sous contrôle tomodensitométrique est très utile dans le traitement des localisations profondes de l'ostéome ostéoïde. Elle permet un repérage précis du nidus et, par conséquent, une résection limitée grâce à un matériel ancillaire adapté. Elle peut autoriser une étude anatomopathologique de la lésion, ce qui n'est pas possible dans les techniques de photocoagulation laser scanno-guidée qui brûlent les tissus et ne permettent pas de faire une étude histologique.

## Conclusion

L'ostéome ostéoïde est une tumeur bénigne, d'origine ostéoblastique. La localisation au talus est rare et de diagnostic souvent tardif. Le traitement chirurgical a bénéficié des progrès de l'imagerie et des techniques de la chirurgie mini-invasive. L'exérèse complète du nidus aboutit à une guérison sans séquelle.
